# Modeling the potential distribution of the energy tree species *Triadica sebifera* in response to climate change in China

**DOI:** 10.1038/s41598-023-51035-x

**Published:** 2024-01-12

**Authors:** Mei Liu, Liyong Yang, Miaomiao Su, Wei Gong, Yibo Liu, Jingxuan Yang, Yi Huang, Cong Zhao

**Affiliations:** 1 Ecological Security and Protection Key Laboratory of Sichuan Province, Mianyang Normal University, Mianyang, 621000 Sichuan China; 2 Tibet Autonomous Region Science and Technology Information Institute, Lhasa, 850000 Tibet China; 3 Sichuan Academy of Forestry Sciences, Chengdu, 610084 Sichuan China; 4 College of Ecology and Environment, Tibet University, Lhasa, 850000 Tibet China; 5 Faulty of Geosciences and Environmental Engineering, Southwest Jiaotong University, Chengdu, 610031 Sichuan China

**Keywords:** Ecological modelling, Ecology, Environmental sciences

## Abstract

As an important woody oilseed species in China, *Triadica sebifera* is not only concerned with the substitution of traditional energy sources, but also plays a considerable role in coping with energy shortages. Accurately predicting the potential geographic distribution of *Triadica sebifera* in China and understanding its ecological needs are crucial for alleviating the energy crisis and effectively implementing energy substitution strategies. In this study, the potential geographic distribution of *Triadica sebifera* in China at contemporary and future periods was predicted based on the distribution data of *Triadica sebifera* in China and the environmental factor variables by Maxent model and ArcGIS software. The combination of important factors governing the potential geographic distribution of *Triadica sebifera* was assessed by the contribution of environmental factor variables. The accuracy of Maxent model's predictions was assessed by AUC values, TSS values and Kappa statistics. The results show that: High AUC and TSS values indicate high accuracy and performance of the model. The crucial environmental factors limiting the potential geographic distribution of *Triadica sebifera* are the temperature factor (mean air temperature of the driest quarter), precipitation factor (precipitation of the coldest quarter, precipitation of the wettest month), and the intensity of human activities (hf). The total suitable area for *Triadica sebifera* is 233.64 × 10^4^ km^2^, primarily located in Yunnan, Sichuan, Hubei, Guizhou, Jiangxi, Guangdong province and Guangxi Zhuang Autonomous Region; its high suitability area is 30.89 × 10^4^ km^2^, accounting for 13.22% of the total suitable area, mainly distributed in Jiangxi, Sichuan and Hunan provinces in the shape of a cake. Under the four typical greenhouse gas emission concentration patterns in the 2050s and 2070s, the areas of high and medium suitable areas for *Triadica sebifera* will increase, while the area of its low suitable area will decrease. However, the total suitable area will remain relatively unchanged. Its potential suitable habitats show a trend of shifting towards lower latitudes and southeast regions. The study predicted the pattern of *Triadica sebifera* under different climate change conditions, which can provide guidance for future cultivation of *Triadica sebifera* as well as for biofuel development and utilization.

## Introduction

In the face of rapid global economic expansion, energy scarcity has emerged as a significant issue to global growth^1–6^. Currently, there is a growing tension between the consumption and supply of traditional energy sources^7,8^, which is having an enormous impact on the global economy and society^9^. The use of traditional energy sources is also an important factor contributing to the deterioration of the human habitat and the frequency of extreme global climate events, which threaten the long-term development of humankind^10,11^. On the other hand, China's per capita oil reserves are significantly below the global average, and it exhibits a pronounced dependence on oil imports^12^. Addressing this severe shortage in oil resources and implementing an oil substitution strategy is crucial for China's sustainable economic development. Biofuel crops are widely recognized as the most important resource for sustainable energy production due to their broad source, environmental friendliness, and renewability^12–14^, and they hold great advantages in oil substitutes^15^.

**Figure 1:**
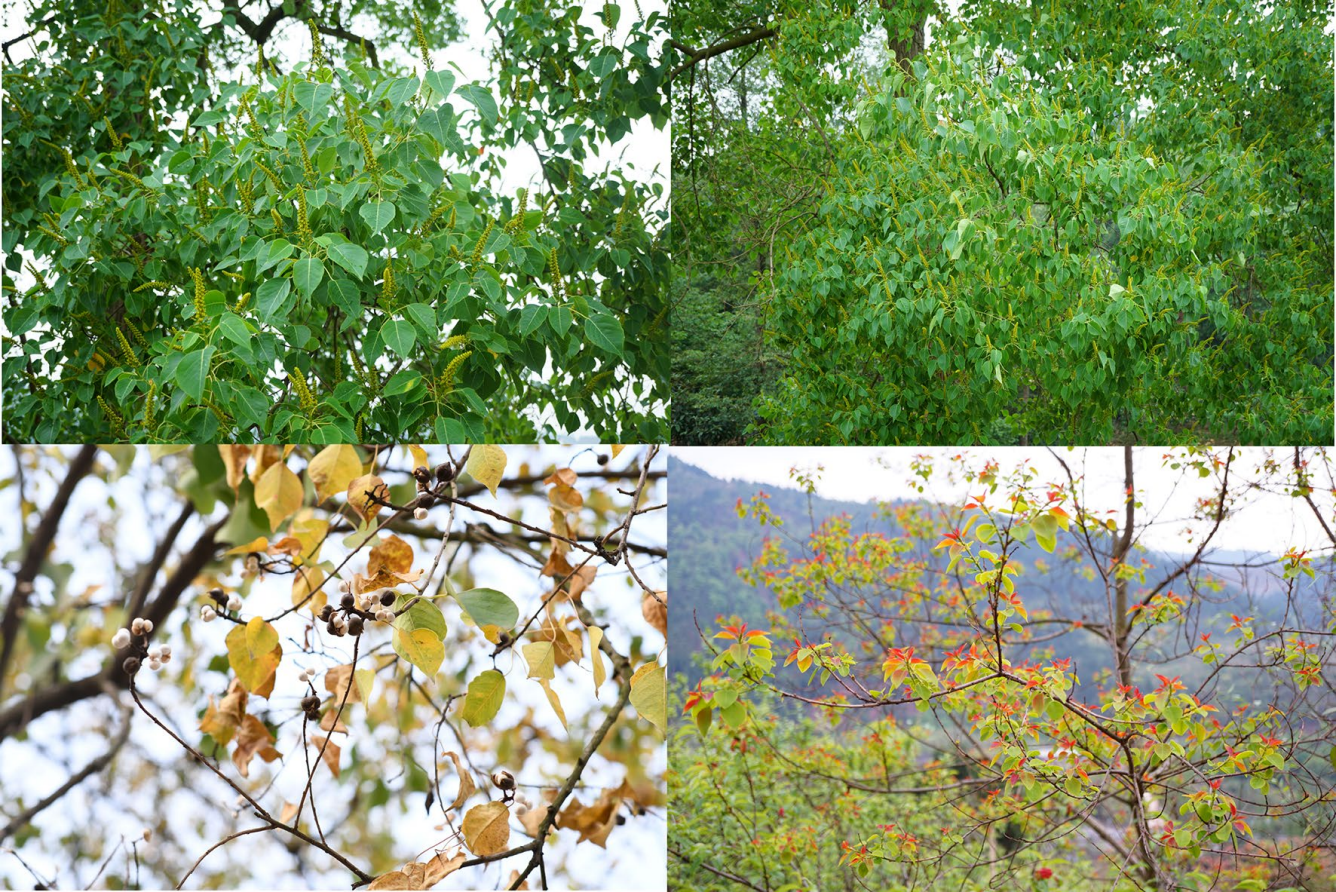
*Triadica sebifera* photographed from wild habitat.

*Triadica sebifera*, also known as the Chinese tallow tree, is a deciduous tree belonging to the genus* Ocimum* in the family *Euphorbiaceae* (Fig. 1). It is an important energy tree species with the title of specialty oil crop^16–18^. *Triadica sebifera* boasts historical mentions within ancient agricultural treatises such as the "Qi Min Yao Shu" and the “Nong Zheng Quan Shu.” It enjoyed widespread cultivation, particularly within China's southwestern and central regions. This tree displays remarkable adaptability, thriving in diverse soil types, including calcareous and acidic soils. Remarkably, it is one of the few oil-bearing crops that can flourish in high-salinity soils^17,19^. Its seeds possess an extraordinarily high oil content, yielding upwards of 40%^19,20^. Given China's limited petroleum resources, the development of woody biomass diesel requires a comprehensive understanding of its ecological requirements and potential distribution areas. As a special class of plant resources, the growth and development of energy tree species are influenced by a variety of factors such as climate, geomorphology, hydrology, and soil type^9,21^. Studies have shown that non-climatic factors dominate only short-term biological changes, and that climate change is the most important factor influencing growth, development and distributional suitability^22–25^. Therefore, it is of great significance to explore the distributional response of *Triadica sebifera* to climate change, which is crucial for its rational cultivation and energy development in China.

To investigate the impact of climate change on the potential geographical distribution of species, Species Distribution Models (SDMs) have been extensively employed^26–29^. Species Distribution Models (SDMs), as the predominant tool for studying species' responses to climate change, have been extensively employed via ecological modeling techniques to predict suitable habitats for energy crops. Such endeavors aim to delineate their ecological prerequisites, offering insights for practical cultivation, such as in the cases of *Miscanthus lutarioriparius*^21^, *Manihot esculenta*^9^, *Brassica napus*^9^, and *Jatropha curcas*^30^. Currently, the mainstream species distribution models include Bioclim, Domain, GARP and MaxEnt, which have been widely used in the fields of plant conservation and utilization, and prevention of invasive pests^31–33^. Due to the differing theoretical foundations of these various models, their simulation effectiveness and predictive performance exhibit substantial variations. Research indicates that the MaxEnt model exhibits the best simulation results among the many species distribution models, and is widely recognized for its robustness and generalizability to incomplete data, which is particularly important in many research areas where data availability is variable. The model is based on the principle of “maximum entropy” and aims to use known distribution points to predict the potential distribution of a target species without making strong assumptions about the completeness of the background data. This is particularly important for species such as *Triadica sebifera*, which are distributed over a wide area but for which habitat data may be incomplete. In addition, MaxEnt's ability to effectively handle nonlinearities and complex interactions in ecological niche modeling allows for more accurate model predictions^34^. *Triadica sebifera*, as an energy crop, the accurate portrayal of its potential habitat is important for guiding practical cultivation. Therefore, it is appropriate to choose MaxEnt model to determine the potential geographic distribution of *Triadica sebifera*.

**Figure 2:**
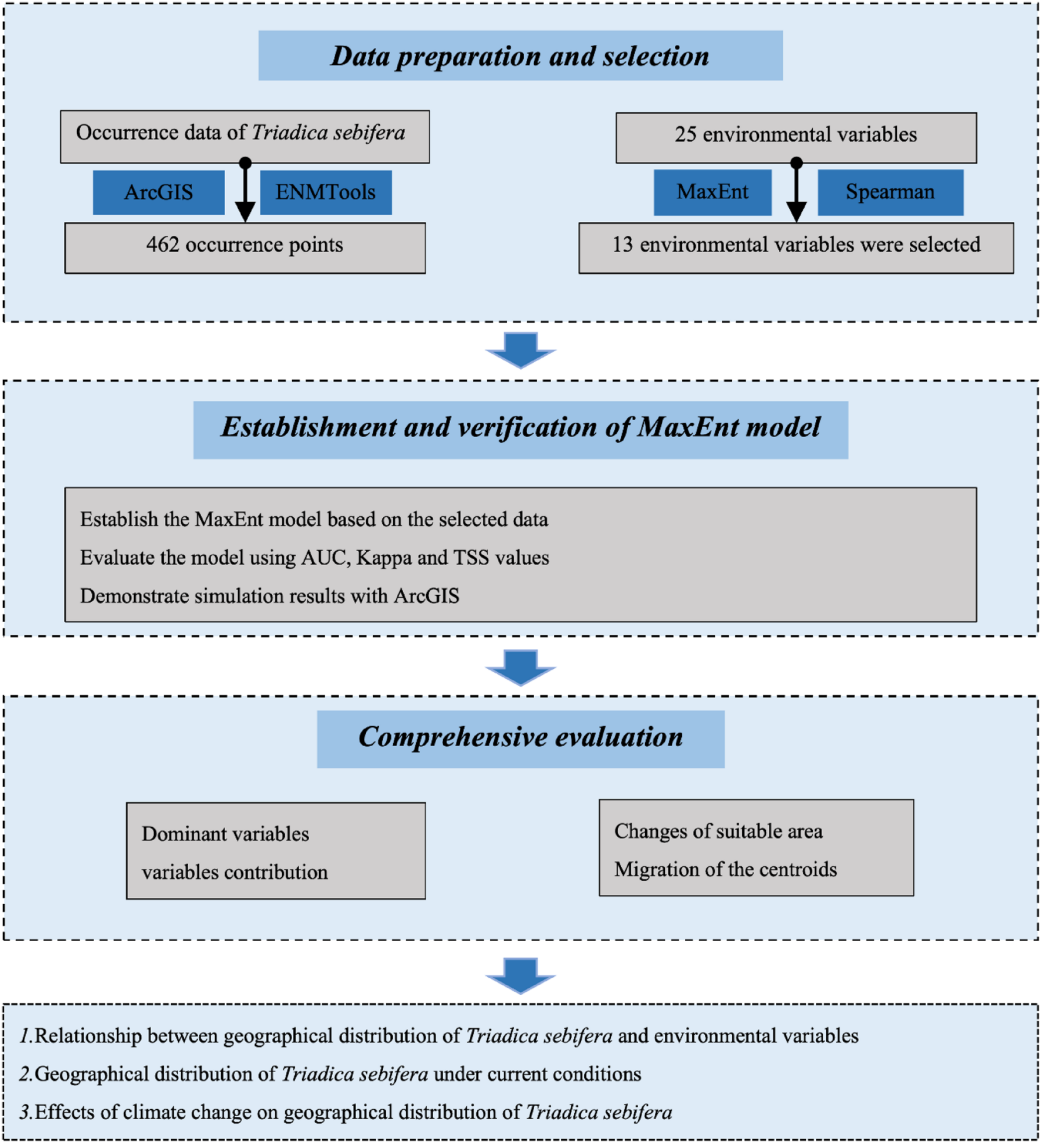
Flowchart displaying the steps of the present study.

Currently, research on energy tree species focuses on the extraction process, but concerning the optimal distribution of energy tree species are notably scarce^9,12^. The cultivation of *Triadica sebifera* also face number of practical difficulties: (1) some of the potential cultivation areas are still unknown; (2) there is an urgent need to know the relationship between the distribution of *Triadica sebifera* and its response to climate change under the stress of global warming. To address the problems above, this research predict the potential geographical distribution zones of *Triadica sebifera* across various time intervals within China. and to provide theoretical references for the cultivation of *Triadica sebifera*, which is described in Fig. 2. The main tasks of this study include: (1) to reveal the current and future distribution patterns of *Triadica sebifera*; (2) illustrating the trends in potential distribution of *Triadica sebifera* under the background of climate change; (3) investigating the relationship between *Triadica sebifera* distribution and its response to climate change.

## Materials and methods

### Source of distribution data for *Triadica sebifera*

**Figure 3:**
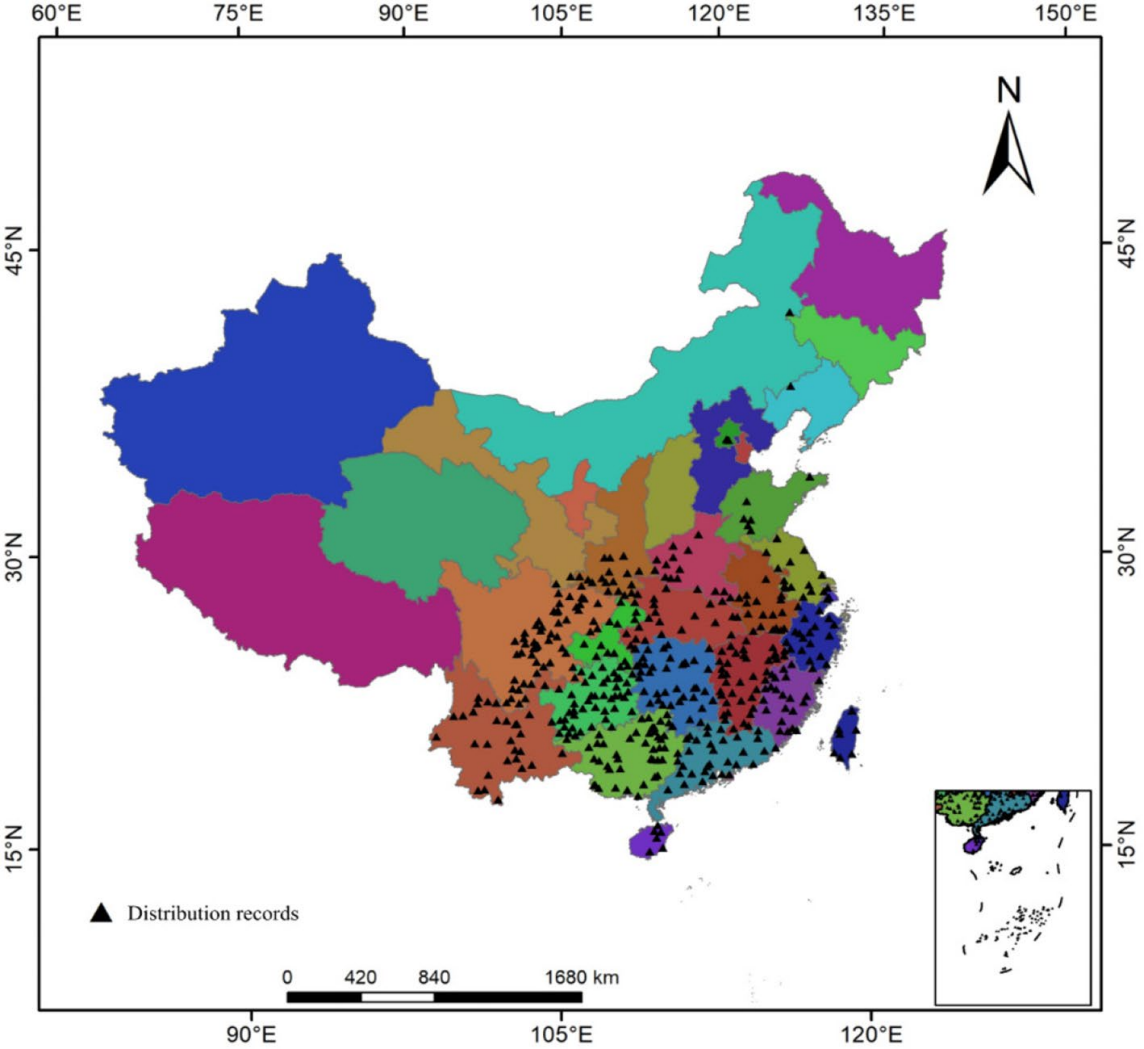
Occurrence records of *Triadica sebifera.*

In this study, the geographic coordinates of *Triadica sebifera* were confirmed and screened by accessing relevant websites such as the National Plant Specimen Resource Center (NPSRC, http://www.cvh.ac.cn/) and removing distribution records with insufficiently specific descriptions and duplicate distributions^26,27^. The concentration of distribution data in an area can overfit the model and bring uncertainty. Therefore, this study utilized the buffer tool in ArcGIS 10.2 to create a buffer zone with a radius of 5 km around each distribution point according to the resolution of the environmental variables, and only one distribution point was kept within 5 km. Eventually 462 distribution points of *Triadica sebifera* were collected and their distribution is shown in Fig. 3.

### Sources of environment variables

Climate data and digital elevation data (DEM, http://www.worldclim.org/) were downloaded from the World Clim database, which contains 19 current 4 future emission scenarios (SSP5-8.5, SSP3-7.0, SSP2-4.5, SSP1-2.6). The current climate database collects detailed meteorological information recorded at weather stations around the world from 1970 to 2000. The future climate data are based on the BCC-CSM2-MR climate system model developed by the National Climate Center. Soil data were obtained from the Harmonized World Soil Database (HWSD V1.2, https://www.fao.org/). UV-B radiation data were downloaded from the Global UV-B Download Radiation Dataset (gIUV, https://www.ufz.de/gluv/index.php). China administrative maps were downloaded from the National Science and Technology Infrastructure System Science Data Center (http://www.geodata.cn). The spatial resolution used to standardize all environmental variables was set to 5 arcmin through ArcGIS software (version 10.2).

**Figure 4:**
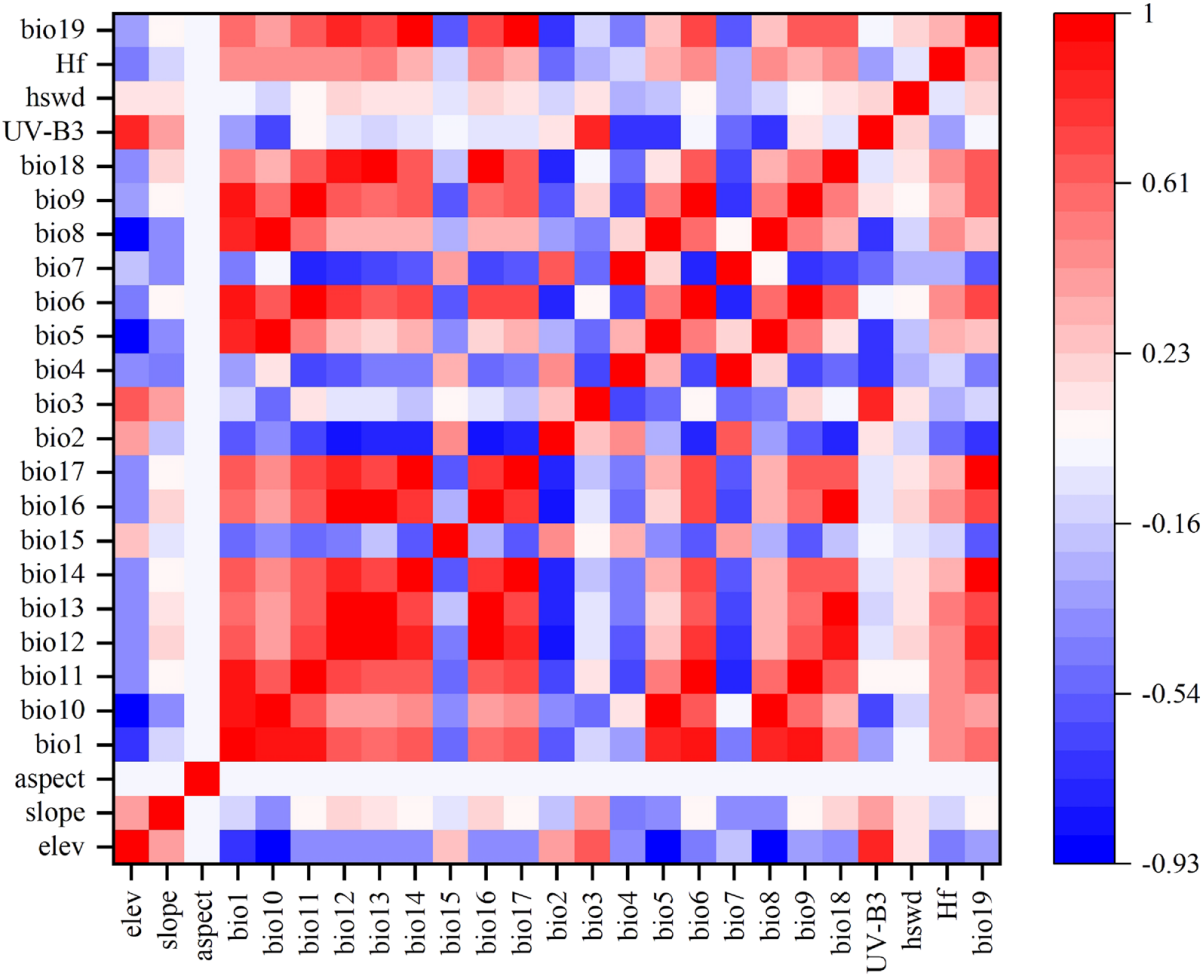
Heat map for correlation analysis of environmental variables.

Since there is a certain correlation between environmental variables, correlation analysis of environmental variables is needed to be used in the MaxEnt model. In this study, Spearman correlation analysis was performed on the environmental factors, and the results are shown in Fig. 4. When the correlation coefficient of two environmental factors is ≥ 0.8, the higher contribution was retained. Twelve environmental factors were finally obtained for MaxEnt modeling (Table 1).

**Table 1:** List of the environmental variables used to develop the model of *Triadica sebifera.*

Variables	Description	Interpretations
Bio6	Min temperature of coldest	Reflecting the effects of temperature extremes
Bio19	Precipitation of coldest	Reflects whether water and heat are synchronized
hf	Intensity of human activity	Reflecting the intensity of human activity
Bio13	Precipitation of wettest month	Reflects extreme moisture conditions
Bio7	Temperature annual range	Reflecting the effects of temperature extremes
slope	Slope	slope
aspect	Aspect	Aspect
Bio5	Max temperature of warmest	Reflects the average temperature and its variability
Bio2	Mean diurnal range (mean of monthly)	Reflecting the characteristics of temperature differences
Bio9	Mean temperature of driest	Reflects whether water and heat are synchronized
UV-B3	Mean UV-B of highest month	Reflects the intensity of ultraviolet radiation
Bio15	Precipitation seasonality (coefficient of variation)	Reflects rainfall and seasonal distribution

### Model construction and evaluation

Distribution point data and environmental factor data for *Triadica sebifera* were imported into MaxEnt software for modeling operations, respectively. 75% of the sample points were randomly selected as the training dataset for modeling, and the remaining 25% of the distribution was used as the test dataset to validate the model. The MaxEnt model requires the user to specify a set of parameters including: the test training (i.e., percentage of locations used for model development and internal testing), the number of background points, the clamp position (i.e., whether to limit the prediction to the variability of the input predicted environmental factors), and the regular multiplier (i.e., to avoid overfitting of the response curve). To evaluate the performance of the parameter configurations, different combinations were selected for trial runs to adjust the optimal parameters of the model. The MaxEnt model regularization level consists of 2 parameters, the modulation multiplicity (RM) and the feature combination (FC). Based on *Triadica sebifera* distribution points and their corresponding environmental factors, the RM was set to 0.5 to 4, respectively. Six sets of FC are set up for optimizing the model parameters to select the best combination of parameters: L (linear feature); LQ (linear feature + quadratic feature), H (hinge feature), LQH (linear feature + quadratic feature + hinge feature), LQHP (linear feature + quadratic feature + product feature) and LQHP (linear feature + quadratic feature + product feature). LQHP (linear features + secondary features + hinge features + product features) and LQHPT (linear features + secondary features + hinge features + product features + threshold features). Finally, the optimized parameters are RM set to 1 and FC to LQHPT.

Species distributions are usually over- or under-estimated when using species distribution models to predict species distributions. Therefore, assessing the accuracy of model simulations using effective evaluation metrics is an important step in determining the accuracy and usability of models. Commonly used theoretical evaluation indexes for species distribution models include overall accuracy, sensitivity, specificity, AUC value, TSS value, Kappa statistic, and different indexes have different judging methods and standards. Among them, AUC, TSS value and Kappa statistic are more widely used. Therefore, to improve the credibility of the validation indexes, this study chooses three assessment methods of AUC, TSS values and Kappa statistic for model accuracy evaluation, and the specific evaluation criteria are shown in Table 2.

**Table 2:** Relationship between the values of kappa, TSS, and AUC and model precision.

Indicator	Excellent	Good	Ordinary	Poor
Kappa	0.85–1.00	0.70–0.85	0.55–0.70	0.00–0.55
TSS	0.85–1.00	0.70–0.85	0.55–0.70	0.00–0.55
AUC	0.90–1.00	0.80–0.90	0.70–0.80	0.00–0.70

### Classification of potentially suitable areas

In the output file, the average of 10 replications was selected as the simulation result for this study. The results were generated as an ASCII raster layer based on the logical value of the probability of species' existence (P), which ranges from 0 to 1. A larger P value indicates a higher likelihood of species' existence. The prediction results were converted into raster format using ArcGIS 10.2 software to classify and visualize suitable habitats. Based on the P-value, the natural discontinuity point method was used to classify the suitable habitat into four grades, and the specific classification criteria are shown in Table 3.

**Table 3:** Range of P-values for different suitable habitat area classes.

P-value range	Suitable habitat area classes
0.6 ≤ P ≤ 1.0	High suitable habitat area
0.3 ≤ P < 0.6	Medium suitable habitat area
0.1 ≤ P < 0.3	Low suitable habitat area
0.0 ≤ P < 0.1	Unsuitable habitat area

## Analysis of results

### Evaluation of model accuracy

**Figure 5:**
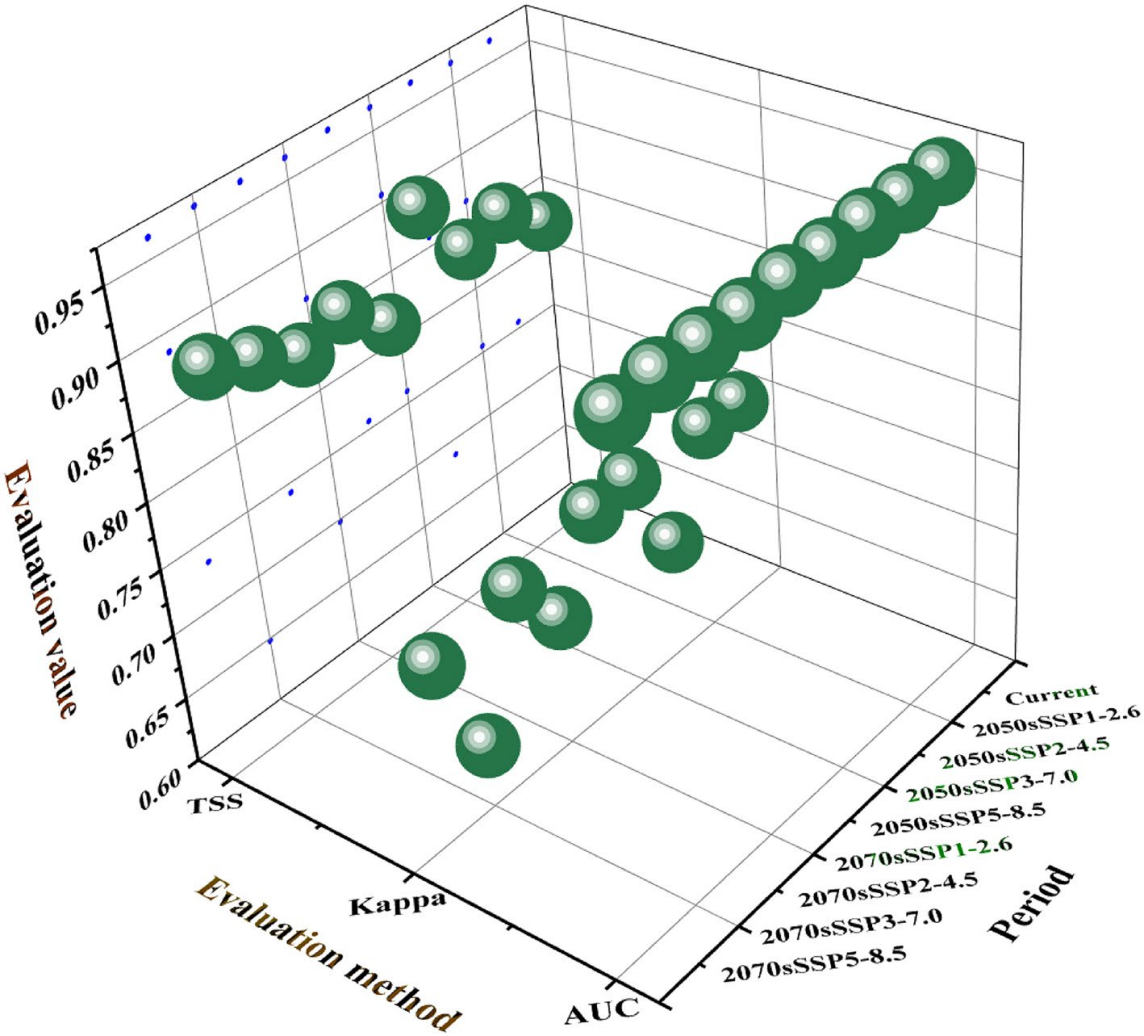
AUC, Kappa and TSS values of MaxEnt.

Simulation prediction of potential habitat of *Triadica sebifera* in China using MaxEnt model based on 462 distribution records. As observed from Fig. 5, the AUC value of the contemporary MaxEnt model established for *Triadica sebifera* stands at 0.965. For the decades of 2050 and 2070, the AUC values under scenarios SSP1-2.6, SSP2-4.5, SSP3-7.0, and SSP5-8.5 range from 0.96 to 0.967. These results suggest that the AUC values of all models significantly surpass that of a random model, all achieving high accuracy rates. The Kappa statistic for the contemporary model is 0.755. The Kappa statistics for the decades of 2050 and 2070 under the scenarios above are in the range of 0.648 to 0.759, indicating that all models' Kappa statistics are superior to "Ordinary," and the predictive results are reliable. Upon comparing the TSS values, the TSS value for the contemporary model is 0.842. The TSS values for the 2050 and 2070 decades range from 0.839 to 0.905 under the scenarios above. The TSS values suggest that the predictive results of all models achieve a "Good" rating or above. All three evaluation indicators demonstrate that the MaxEnt model for *Triadica sebifera* established in this study is reasonably configured, reliable, and suitable for subsequent analysis.

### Environmental factors affecting the potential geographic distribution of *Triadica sebifera*

**Figure 6:**
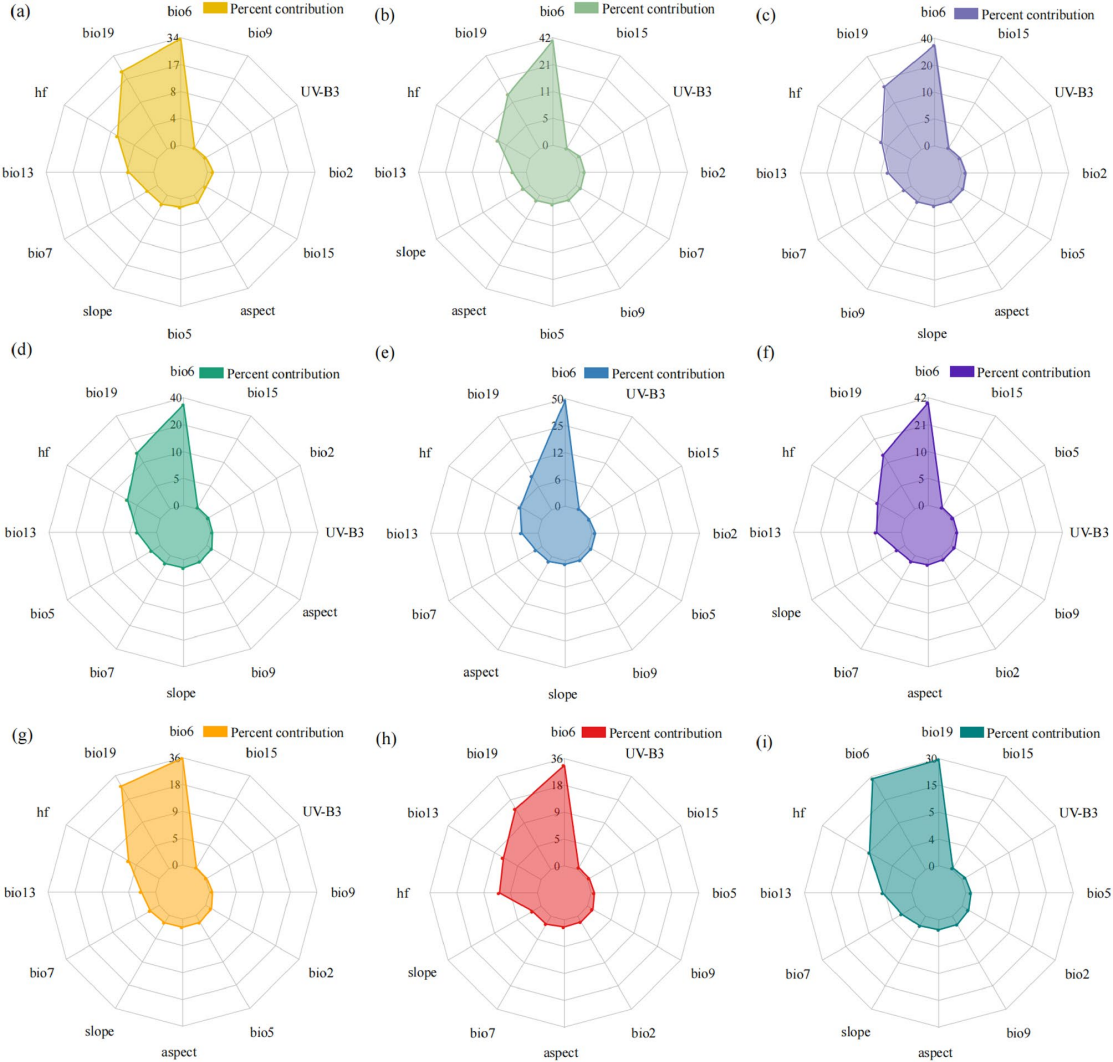
Environmental variables and their contributions of *Triadica sebifera*, (**a**) 2050s, SSP1-2.6; (**b**) 2050s, SSP2-4.5g; (**c**) 2050s, SSP3-7.0; (**d**) 2050s SSP5-8.5; (**e**) 2070s, SSP1-2.6; (**f**) 2070s, SSP2-4.5g; (**g**) 2070s, SSP3-7.0; (**h**) 2070s SSP5-8.5; (**i**) current.

Currently, academics lack a uniform methodology for determining the number of major factors, with most advocating a contribution-based assessment. But the choice of degree is subjective, leading to the emergence of different criteria. This study selects the top four environmental factors in terms of contribution rate as the dominant environmental factor combinations. Quantitative statistics for environmental variables in terms of contribution rate (Fig. 6) show that among the modeled environmental factors, Min Temperature of Coldest (Bio6), Precipitation of Coldest (Bio19), Intensity of Human Activity (hf) and Precipitation of Wettest Month (Bio13) contributed much more than the other variables in the top four positions in any period and in any concentration emission context, which were used as the dominant environmental factor combination in this study.

### Current potential geographic distribution of *Triadica sebifera*

**Figure 7:**
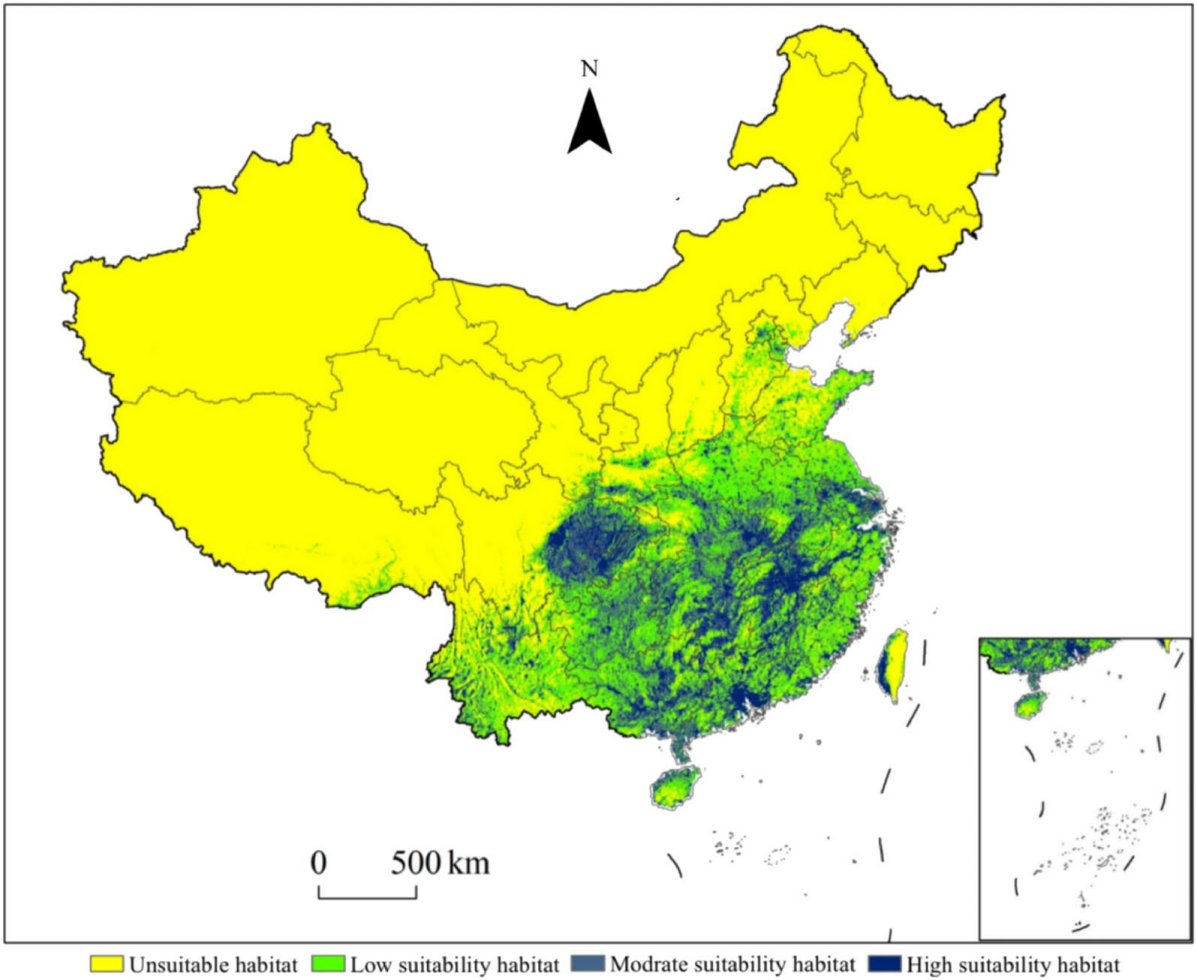
Current geographical distributions of *Triadica sebifera.*

The current potential distribution area of *Triadica sebifera* was simulated as shown in Fig. 7. The total suitable area of *Triadica sebifera* was 233.64 × 10^4^ km^2^ (Fig. 9), which is mainly located in Yunnan, Hubei, Guizhou and Jiangxi Province, and the eastern part of Sichuan Province and Guangdong Province and Guangxi Zhuang Autonomous Region. The high suitable area of *Triadica sebifera* was 30.89 × 10^4^ km^2^, accounting for 13.22% of the total suitable area (Fig. 9), part of which was mainly distributed in Jiangxi, Hunan, Guangdong Province and Sichuan Province, and Guangxi Zhuang Autonomous Region and other provinces (Fig. 7). The medium suitable area of *Triadica sebifera* was 77.26 × 10^4^ km^2^, accounting for 33.07% of the total suitable area (Fig. 9), mainly distributed in Hunan, Sichuan, Hubei, Guizhou, Jiangxi, Guangdong and Yunnan Province and Guangxi Zhuang Autonomous Region (Fig. 7). The low suitable area of *Triadica sebifera* was 125.49 × 10^4^ km^2^, accounting for 53.71% of the total suitable area (Fig. 9), and was mainly distributed in Yunnan, Henan, Guizhou, Hubei, Anhui and Hunan Province, and Guangxi Zhuang Autonomous Region, (Fig. 7).

In summary, the Maxent model predictions show that the potential geographic distribution of *Triadica sebifera* is very large, and the potential geographic distribution range is much larger than the modern geographic distribution range of *Triadica sebifera* as described by the Chinese Plant Wisdom (http://www.iplant.cn) (Fig. 7).

### Future potential geographical distributions of *Triadica sebifera*

**Figure 8:**
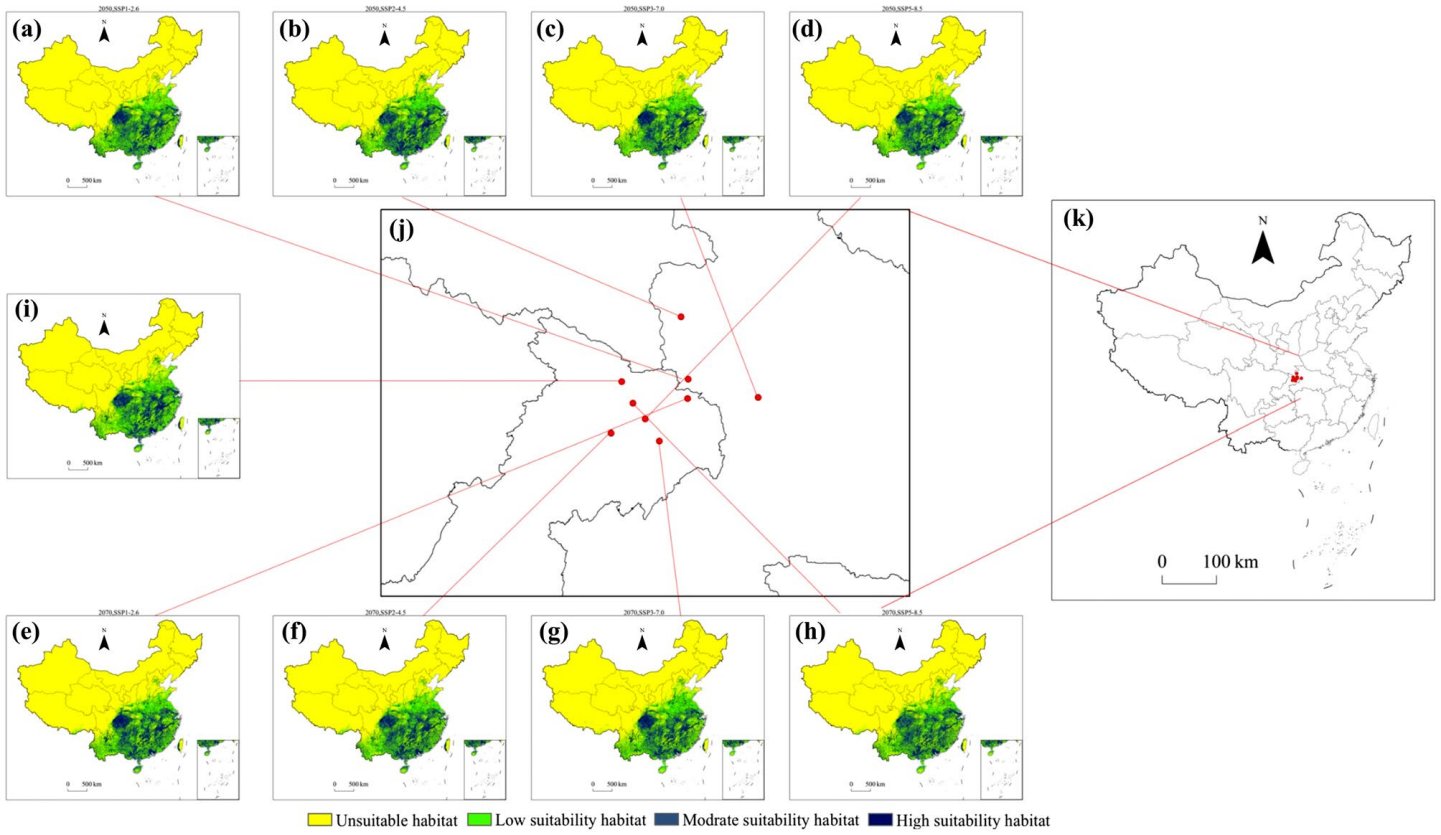
Potential geographical distribution of *Triadica sebifera* in the 2050s and 2070s predicted using MaxEnt and Variations of the centroids of total suitability habitat area of *Triadica sebifera* under climate change scenarios. (**a**) 2050s, SSP1-2.6; (**b**) 2050s, SSP2-4.5g; (**c**) 2050s, SSP3-7.0; (**d**) 2050s SSP5-8.5; (**e**) 2070s, SSP1-2.6; (**f**) 2070s, SSP2-4.5g; (**g**) 2070s, SSP3-7.0; (**h**) 2070s SSP5-8.5; (**i**) current; (**j,k**) center of gravity of a suitable habitat.

**Figure 9:**
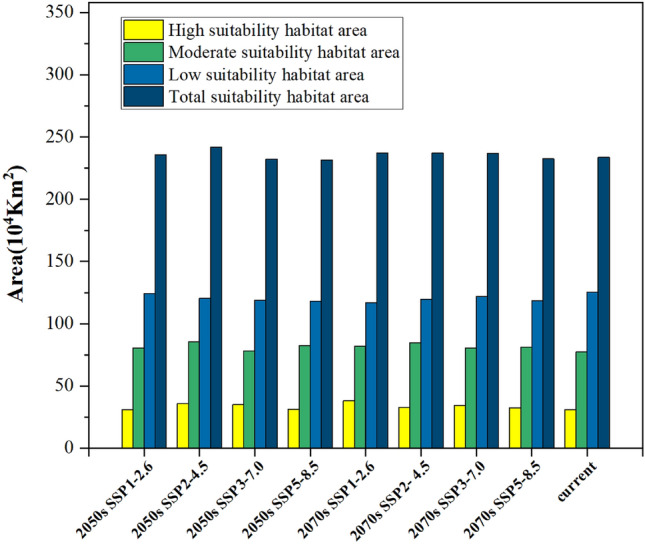
Suitable areas for *Triadica sebifera* under different climate change scenarios.

The model predicted the potential suitable habitat areas for *Triadica sebifera* under four (SSP1-2.6, SSP2-4.5, SSP3-7.0 and SSP5-8.5) different emission scenarios in 2050s and 2070s. Potential suitable areas for *Triadica sebifera* under climate change scenarios and changes in the center of gravity of suitable habitats based on modern climate conditions and future climate change scenarios were obtained (Fig. 8). Compared with the current climate conditions, the area of high and medium suitable zones of *Triadica sebifera* increased and the area of low suitable zones decreased under all four scenarios in the 2050s and 2070s, with very little change in the areas of total suitable zones. Only the SSP3-7.0 emission scenario at the 2050s, the SSP3-7.0 emission scenario at the 2050s, and the SSP5-8.5 emission scenario at the 2070s showed a slight decrease in total suitable area in both time periods, and the total suitable area increased in all other platoon scenarios (Figs. 8 and 9).

Compared to the current distribution, under the SSP5-8.5 emission scenario for the 2050s period, the total suitable area decreased by 0.89%, the high suitable area increased by 1.52%, the medium suitable area increased by 6.52%, and the low suitable area decreased by 6.05% (Fig. 9). Under the SSP3-7.0 emission scenario at the 2050s period, the total fitness zone area decreased by 0.60%, the high fitness zone area increased by 13.37%, the medium fitness zone area increased by 1.20%, and the low fitness zone area decreased by 5.15% (Fig. 9). Under the SSP2-4.5 emission scenario at the 2050s period, the total suitable area increased by 3.50%, the high suitable area increased by 16.12%, the medium suitable area increased by 10.59%, and the low suitable area decreased by 3.98% (Fig. 9). Under the SSP1-2.6 emission scenario at the 2050s period, the total suitable area increased by 0.83%, the high suitable area increased by 0.10%, the medium suitable area increased by 4.06%, and the low suitable area decreased by 0.99% (Fig. 9).

Compared to the current distribution, under the SSP5-8.5 emission scenario for the 2070s period, the total suitable area decreased by 0.55 percent, the high suitable area increased by 5.41 percent, the medium suitable area increased by 5.28 percent, and the low suitable area decreased by 5.60 percent (Fig. 9). Under the SSP3-7.0 emissions scenario for the 2070s period, the total suitable area increased by 1.36 percent, the high suitable area increased by 10.68 percent, the medium suitable area increased by 4.25 percent, and the low suitable area decreased by 2.72 percent (Fig. 9). Under the SSP2-4.5 emissions scenario for the 2070s period, the total suitable area increased by 1.49%, the high suitable area increased by 6.38%, the medium suitable area increased by 9.51%, and the low suitable area decreased by 4.66% (Fig. 9). Under the SSP1-2.6 emission scenario for the 2070s period, the total suitable area increased by 1.47%, the high suitable area increased by 23.11%, the medium suitable area increased by 5.98%, and the low suitable area decreased by 6.67% (Fig. 9).

The centers of gravity of suitable habitats for *Triadica sebifera* under modern climate conditions and future climate change scenarios reveal the trajectories and trends of its potential suitable habitats. As shown in Fig. 8, the center of gravity of the potential suitable habitat of *Triadica sebifera* tends to shift to the southeast and low latitude under all four emission scenarios in 2050 and 2070. Under the 2050 SSP3-7.0 emission scenario, the trend of shifting the center of gravity of potentially suitable habitat for *Triadica sebifera* is the most significant, and in general, the magnitude of the shift is not significant.

In summary, the center of gravity of *Triadica sebifera*’s suitable areas and changes in the center of gravity under the climate change scenarios are not significant. Potential suitable habitats for *Triadica sebifera* do not change much under different concentration emission scenarios in the 2050s and 2070s. Some low and medium suitable areas were converted to high suitable areas, and some low and unsuitable areas were converted to medium suitable areas. Still, some suitable areas disappeared (Figs. 8 and 9).

## Discussion

### Effects of environmental variables on the potential geographic distribution of *Triadica sebifera*

The important environmental factors limiting the potential geographic distribution of *Triadica sebifera*, as predicted by the MaxEnt model, are the temperature factor (mean air temperature of the driest quarter), the precipitation factor (precipitation of the coldest quarter, precipitation of the wettest month), and the intensity of human activities (hf). The probability of presence of *Triadica sebifera* increased to some extent as the mean temperature of the driest quarter increased, which may be related to the fact that *Triadica sebifera* prefers hot, humid, and sunny temperatures. Pattison and Mack showed that minimum temperature and limited precipitation were the main climatic constraints to *Triadica sebifera* in the eastern and western United States, respectively^35^. The increase in precipitation in the coldest season somewhat reduced the probability of *Triadica sebifera*’s presence; with the increase in precipitation in the wettest month, the probability of *Triadica sebifera*’s presence increased. Gu et al. explored the correlation between environmental factors and seed yield and quality of *Triadica sebifera*. They showed that as the precipitation increased in the coldest season, the seed yield of *Triadica sebifera* was lower, and the quality was poorer^35^. Studies by Wang^36^ and Rong et al.^37^ also showed that temperature and precipitation factors are important environmental factors limiting the potential geographic distribution of *Triadica sebifera*, which corroborates with the present study. The intensity of human activity is strongly linked to the probability of presence of *Triadica sebifera*, which is also related to the fact that humans have been utilizing *Triadica sebifera*. Related studies have shown that humans have been utilizing *Triadica sebifera* since prehistoric times^38^, so human activities should also be considered in cultivating *Triadica sebifera*.

This study predicts the potential geographic distribution of *Triadica sebifera* in China and identifies the climatic factors that limit the potential geographic distribution of *Triadica sebifera*. Expansion of the study area may make the range of environmental factors limiting the growth of *Triadica sebifera* change. Other environmental factors, such as vegetation cover and other data, influence the potential geographic distribution of *Triadica sebifera*. Since it is impossible to accurately predict the vegetation cover in China in the future, it was not included in the prediction of the potential geographic distribution of *Triadica sebifera*. Therefore, some potential geographic distribution areas in this study may not be suitable for *Triadica sebifera* and must be adapted to the local hydrogeological conditions when applied in practice. However, the results of this study are the first step of macro-planning, which is crucial for the scientific management and exploitation of *Triadica sebifera*.

### Changes in the potential geographic distribution of *Triadica sebifera* under future climate change scenarios

**Figure 10:**
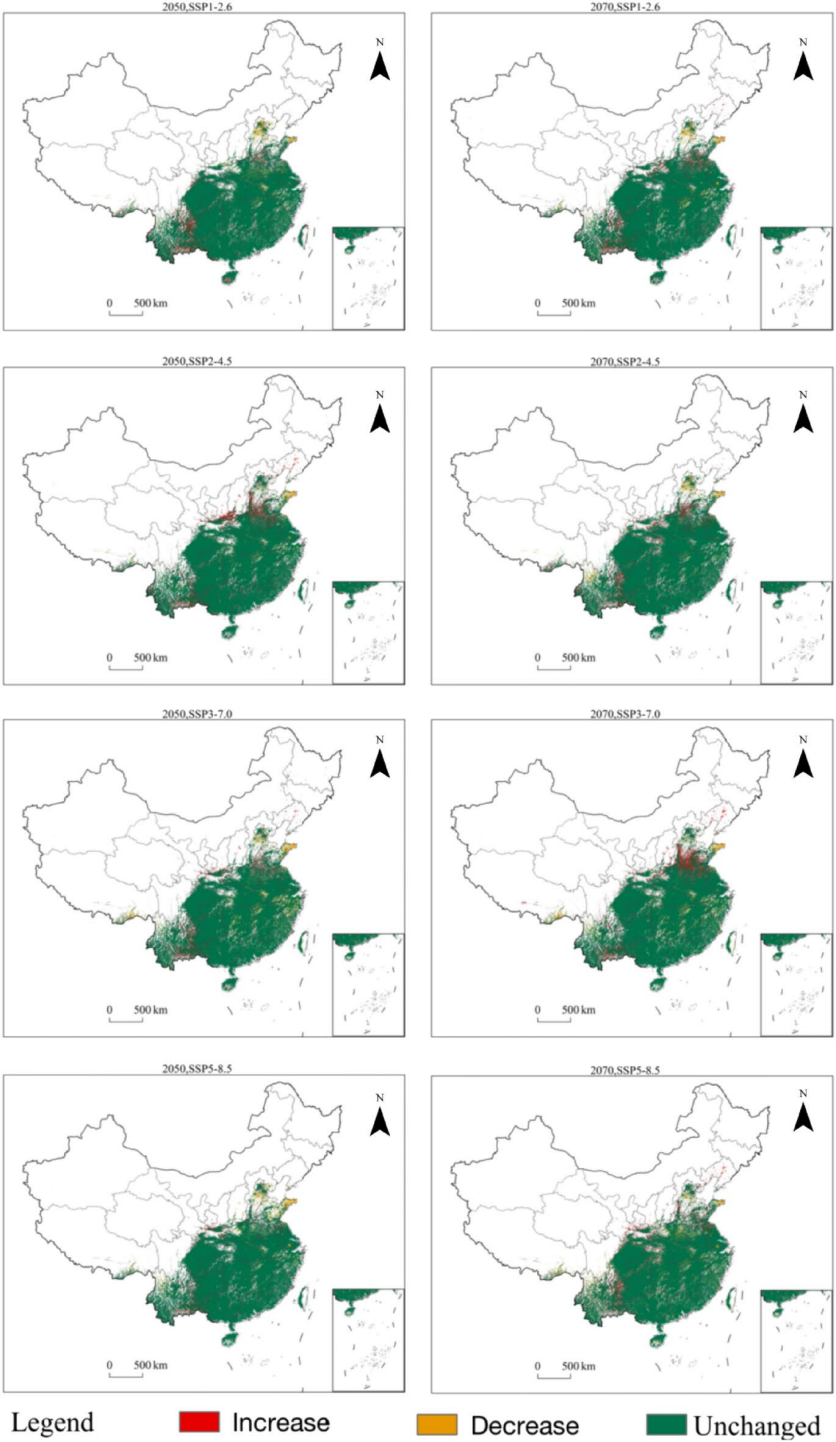
Changes in the potential geographical distribution of *Triadica sebifera* under climate change scenarios in the future.

The potential geographic distribution of *Triadica sebifera* in China under future climate change scenarios was predicted using the MaxEnt model based on the environmental factors under four emission scenarios in 2050 and 2070 combined with modern climate conditions (Fig. 8). The results were spatially overlaid and analyzed in ArcGIS to obtain changes in the potential distribution of *Triadica sebifera* under future climate change scenarios (Fig. 10).

Predictive outcomes indicate that, under four emission scenarios in 2050 and 2070, the potential geographic distribution of *Triadica sebifera* displays negligible overall alterations compared to its distribution under modern climate conditions, exhibiting a minor increasing trend. Only under the 2070’s SSP5-8.5 emission scenario and the 2050’s SSP5-8.5 and SSP3-7.0 emission scenarios does the total area of suitable habitats present a slight downward trend (Fig. 10). The results suggest that temperature and precipitation constrain the survival of *Triadica sebifera*, probably because the temperature and precipitation under the high emission scenario exceed the thresholds suitable for the survival of *Triadica sebifera*, which decreases the probability of its survival in some areas. Under the other emission scenarios, the temperature and precipitation were within the range suitable for the survival of *Triadica sebifera*, which resulted in an increasing trend of potential suitable habitat for *Triadica sebifera*. The center of gravity of potentially suitable habitat shifted to the southeast and lower latitudes (Fig. 7), but to a lesser extent, possibly implying that some areas become unsuitable for *Triadica sebifera* under future climate change scenarios. There will be areas suitable for *Triadica sebifera*, but the area of change is not large. Thomas et al., on the extinction risk of organisms in an area covering 20% of the Earth's surface, suggests that 15–37% of species will be at risk of extinction under the medium emissions scenario in 2050. Other species are at less risk of extinction, and some species will benefit from warming, suggesting that the effects of warming on the potential geographic distribution of species are twofold and that not all species will be at risk of extinction or will benefit equally from climate change^39^.

**Figure 11:**
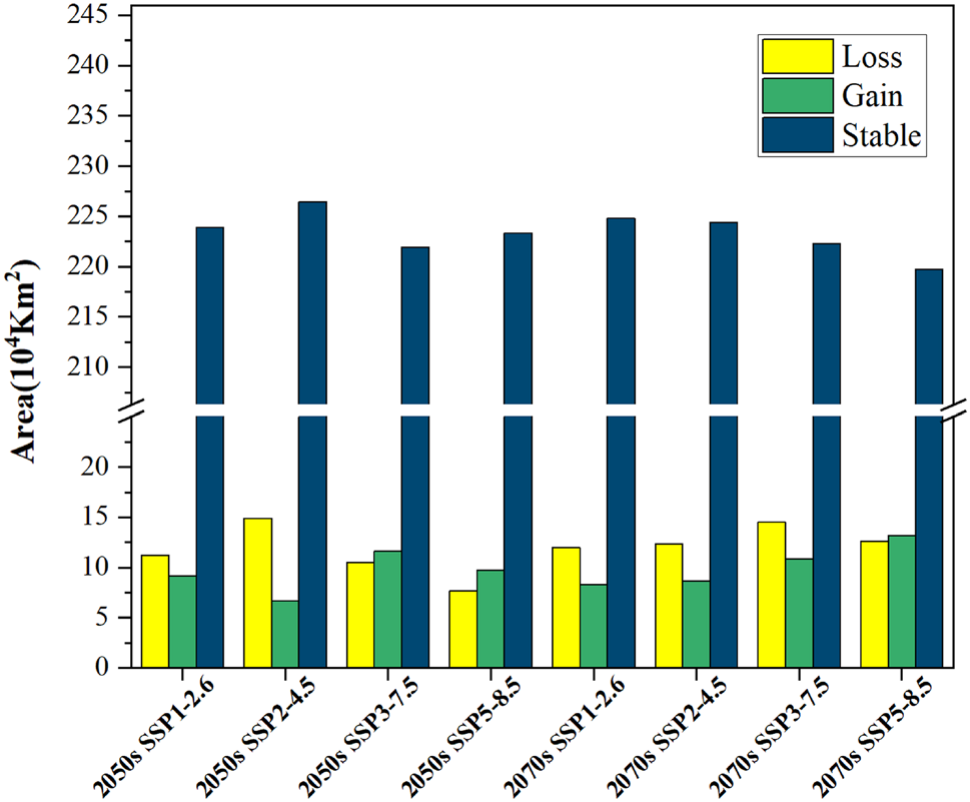
Changes of *Triadica sebifera* in suitable habitat areas in the future.

Under the four emission scenarios in 2050 and 2070, suitable habitat for *Triadica sebifera* increased to different degrees in central and southern China and decreased to different degrees in high latitude and high altitude areas in northern and northwestern China, with little change in general (Fig. 10). Under the emission scenario SSP2-4.5 in 2050, the area of habitat loss for *Triadica sebifera* is minimal, encompassing a region of 6.67 × 10^4^ km^2^. In contrast, under the SSP5-8.5 emission scenario in 2070, the habitat loss for *Triadica sebifera* extends to its maximum, a substantial area of 13.19 × 10^4^ km^2^ (Fig. 11). The principal regions of habitat loss for *Triadica sebifera* are found in Yunnan Province, Shandong Province, Henan Province, Guangxi Zhuang Autonomous Region, and Sichuan Province. This mainly manifests as losing low-suitability habitats, with some regions transitioning between medium and high-suitability habitats (Figs. 8 and 10).

Under the SSP5-8.5 emission scenario in 2050, the least expansion of suitable habitats for *Triadica sebifera* is observed, covering an area of 7.68 × 10^4^ km^2^. Conversely, under the SSP2-4.5 emission scenario in 2050, the largest expansion of suitable habitats for *Triadica sebifera* is noted, encompassing an area of 14.89 × 10^4^ km^2^ (Fig. 11). The expansion areas of *Triadica sebifera* predominantly lie in provinces of Yunnan, Shandong, Shaanxi, Sichuan, and Hebei (Fig. 10). A significant reason for the increased suitability of *Triadica sebifera* in Yunnan, Shaanxi, and Sichuan provinces is that the Qinling and Bashan regions of China are located on the northern edge of the subtropics, serving as the convergence zone of subtropical and warm temperate climates. The immense barrier of the Qinling-Bashan region impedes the northward progression of the southeast monsoon and the southern invasion of cold northern air, thus providing an ideal environment for various species to thrive and multiply^40^. As indicated by the Shared Socioeconomic Pathways scenarios proposed in the Sixth Assessment Report (AR6). (2021) by the Intergovernmental Panel on Climate Change, global warming trends and temperature increases are becoming more pronounced^41^. Given that temperature significantly impacts *Triadica* *sebifera*'s growth, elevated temperatures resulting from high emission scenarios could potentially lead to the expansion of unsuitable habitats for *Triadica sebifera*. This could explain the greatest loss of habitat under high-emission scenarios and shifts in the distribution of centroids. The influence of global warming on the potential geographical distribution of species manifests primarily in the migration of species to different latitudes or altitudes and the expansion or contraction of potential geographical distribution areas. The trends observed in this study regarding the migration of potentially suitable habitats for *Triadica sebifera* under future climate change conditions align with these characteristics^42^.

Climate change indirectly affects the population and distribution characteristics of *Triadica sebifera* by directly affecting the ecosystem. In addition to the important impacts of climate change on the potential geographic distribution of *Triadica sebifera*, irrational agricultural development, the rise of tourism activities, hydropower development, and other industrial behaviors may also lead to changes in the geographic distribution of *Triadica sebifera*. In this study, only two time periods, 2050 and 2070, were used for the environmental factor variables, so in future studies on the response of the potential geographic distribution of species to climate change, multiple study periods can be chosen to derive the overall trend of the potential geographic distribution of the study population.

## Conclusion

This study identifies the dominant environmental factors affecting the potential geographic distribution of *Triadica sebifera*, including temperature (mean air temperature in the driest season), precipitation (precipitation in the coldest season and precipitation in the wettest month), and the intensity of human activities. Under modern climatic conditions, this plant is mainly found in provinces and regions south of the Yellow River and north of Shaanxi and Gansu. Projections for four climate scenarios in the 2050s and 2070s indicate that the area of highly and moderately suitable habitat areas for *Triadica sebifera* will increase, while the area of low suitable habitat areas will decrease. Although there is little change in overall suitable habitat areas, there is a trend toward a slight shift toward lower latitudes and the Southeast. These findings have important applications for predicting and planning the cultivation of *Triadica sebifera* and the development of biofuels, and are equally instructive in the fields of ecological restoration, biodiversity conservation, and the sustainable development of energy crops.

*Triadica sebifera* is not only an important woody oilseed species, but also has ornamental and medicinal values. Therefore, its cultivation and utilization in the field of bioenergy is promising. Future research should focus on the following aspects: first, long-term field trials to validate the model predictions of this study, especially to the potential for biofuel production. Second, in-depth exploration of the long-term impacts of climate change on *Triadica sebifera* and its associated ecosystem services. In addition, the growth performance and oil yield of *Triadica sebifera* under different cultivation conditions are investigated to determine optimal cultivation management strategies. Finally, considering the potential risk of plant invasions, future research should also assess the ecological impacts of the spread of *Triadica sebifera* and develop effective risk management measures. These studies will not only help to maximize the economic value of *Triadica sebifera* as a bioenergy source as well as other uses, but also ensure environmental protection and ecological balance.

## Data Availability

All data included in this study are available upon request by contact with the corresponding author.
